# Morphine Withdrawal-Induced Hyperalgesia in Models of Acute and Extended Withdrawal Is Attenuated by *l*-Tetrahydropalmatine

**DOI:** 10.3390/ijms24108872

**Published:** 2023-05-17

**Authors:** Daria Oleinichenko, Soyon Ahn, Ru Song, Terrance P. Snutch, Anthony G. Phillips

**Affiliations:** 1 Department of Psychiatry, University of British Columbia, Vancouver, BC V6T 2A1, Canada; 2 Djavad Mowafaghian Centre for Brain Research, University of British Columbia, Vancouver, BC V6T 1Z3, Canada; 3 Michael Smith Laboratories, University of British Columbia, Vancouver, BC V6T 1Z4, Canada

**Keywords:** Von Frey test, *l-*tetrahydropalmatine, rat, opioid detoxification, substance use disorder, pain, withdrawal-induced hyperalgesia

## Abstract

Effective pain control is an underappreciated aspect of managing opioid withdrawal, and its absence presents a significant barrier to successful opioid detoxification. Accordingly, there is an urgent need for effective non-opioid treatments to facilitate opioid detoxification. *l*-Tetrahydropalmatine (*l*-THP) possesses powerful analgesic properties and is an active ingredient in botanical formulations used in Vietnam for the treatment of opioid withdrawal syndrome. In this study, rats receiving morphine (15 mg/kg, i.p.) for 5 days per week displayed a progressive increase in pain thresholds during acute 23 h withdrawal as assessed by an automated Von Frey test. A single dose of *l*-THP (5 or 7.5 mg/kg, p.o.) administered during the 4th and 5th weeks of morphine treatment significantly improves pain tolerance scores. A 7-day course of *l*-THP treatment in animals experiencing extended withdrawal significantly attenuates hyperalgesia and reduces the number of days to recovery to baseline pain thresholds by 61% when compared to vehicle-treated controls. This indicates that the efficacy of *l*-THP on pain perception extends beyond its half-life. As a non-opioid treatment for reversing a significant hyperalgesic state during withdrawal, *l*-THP may be a valuable addition to the currently limited arsenal of opioid detoxification treatments.

## 1. Introduction

Increased access to potent pain-relieving opioids is linked to an unprecedented rise in opioid use disorder (OUD) and opioid-related deaths [1,2]. Opioid detoxification, a process necessary to overcome physical dependence on opioids, is a critical first step in transitioning to long-term management of OUD. However, abrupt discontinuation triggers opioid withdrawal syndrome (OWS), a debilitating condition that includes severe pain, negative affect/dysphoria, and somatic signs that last for several days to many weeks [3]. For individuals with a history of chronic opioid use, which often includes prior unsuccessful attempts to abstain from these drugs, the urge to avoid OWS is a primary cause of failure to complete detoxification and therefore is a powerful driving factor in maintaining opioid use [3,4]. Current protocols for detoxification often involve substitution therapy with long-acting opioids (e.g., methadone, buprenorphine), which introduce problems related to side effects and diversion to illicit use. Accordingly, there is great interest in non-opioid compounds that may have efficacy in facilitating detoxification from opioids. 

The misuse of opioids commonly arises from the need to control pain, but ironically, their continued use leads to hypersensitivity to pain, a phenomenon known as opioid-induced hyperalgesia (OIH) or opioid tolerance [5]. Clinically, OIH is evident in individuals with OUD currently on methadone maintenance and those prescribed opioids for perioperative pain [6,7,8]. Adding insult to injury, we now know that opioid withdrawal, whether during voluntary detoxification, lack of access to an illicit drug supply, or naloxone-precipitated withdrawal, also elicits a heightened state of pain perception, called “opioid withdrawal-induced hyperalgesia” [9,10]. Animal studies by Koob and others demonstrate that opioid withdrawal-induced hyperalgesia is a phenomenon that remains stable for several weeks, with the involvement of both central and peripheral neural substrates [11,12,13,14,15]. Thus, enhanced pain perception, which begins during acute withdrawal and persists into protracted abstinence [9,10], likely contributes to an aversive state that drives drug-seeking behavior [16]. Recent clinical work confirms a positive correlation between the severity of pain and the frequency at which patients report pain coping as their motivation for drug-seeking [17]. As yet, pain management remains an undertreated aspect of OUD, particularly following opioid detoxification [16,18].

Medicinal plant research has been gaining prominence as many herbs used in traditional Asian medical practice have been proven to be efficacious against a spectrum of nervous system disorders. A recent review by Jaffal and Abazid highlights more than a dozen medicinal plants that have demonstrated potential as remedies against substance misuse [19]. A notable candidate, *Corydalis Yanhusuo*, is well recognized in China for its analgesic properties and its efficacy in an array of preclinical assays for allodynia [20]. A database analysis of traditional Chinese medicine used for the treatment of substance use disorders highlights *Corydalis Yanhusuo* among the most frequently used medicinal herbs [21]. Over 100 compounds, approximately 60 of which are pharmacologically active alkaloids, have been isolated from *Corydalis Yanhusuo* [22]. *Corydalis Yanhusuo* is a tetrahydroprotoberberine (THPB)-rich plant, which is one class of compounds that hold promise as novel non-opioid treatments for OUD [23]. It is widely recognized that *l*-tetrahydropalmatine (*l*-THP), a THPB alkaloid also found in several medicinal plants of the *Stephania* genus [24,25,26,27], is one of the most prominent active constituents of *Corydalis Yanhusuo*. Indeed, the analgesic and substance misuse remedying properties of the extracts are primarily attributed to *l*-THP [28,29], as well as its active metabolite, *l*-isocorypalmine (*l*-ICP), which is another THBP constituent of *Corydalis Yanhusuo* with a similar pharmacodynamic profile [30].

In China, *l*-THP is one of the most extensively investigated phytochemicals for the treatment of brain disorders, including schizophrenia, addiction, and pain [23,31]. Both *Corydalis Yanhusuo* extract and *l*-THP show efficacy in treating addiction in animal models. *l*-THP inhibits self-administration and relapse in animal models of cocaine misuse [32,33] when administered alone, and it exhibits even greater efficacy when used in combination with naltrexone, an opioid antagonist [34]. Similar effects of *l*-THP are seen in an animal model of methamphetamine dependence [35]. *l*-ICP, the active metabolite of *l*-THP, is also effective in attenuating cocaine-induced conditioned place preference [36]. *l*-THP has been evaluated for efficacy in several models of OUD and is shown to reduce self-administration and relapse rates in a rat heroin abuse model [37] and prevent expression of morphine-induced conditioned place preference [38]. Similar effects are seen with opioids, known for their high abuse potential. *l*-THP prevents the development of locomotor sensitization to oxycodone [39] and blocks fentanyl-induced conditioned place preference [40]. Recently, Alhassen et al. have demonstrated in preclinical studies that *Corydalis Yanhusuo* extract improves morphine analgesia while preventing the development of morphine tolerance. The extract also attenuates the rewarding properties of morphine and reduces the presentation of somatic signs of withdrawal, indicating its potential for preventing the development of dependence [41]. Clinically, *l*-THP also reduces craving and improves treatment outcomes in heroin users undergoing detoxification [42]. Orally administered *l*-THP in cocaine-dependent patients and drug-free volunteers is bioavailable, safe, and well-tolerated [43,44]. 

Of particular relevance to the present study, *l*-THP shows efficacy in neuropathic and inflammatory models of neuropathic pain, likely through a combination of spinal [45] and supraspinal mechanisms [46,47,48]. The wide-ranging medicinal properties of *l*-THP (e.g., analgesic, anxiolytic, anti-inflammatory, and relief of gastrointestinal illness) [23,31,36,49,50] are attributed in part to dopamine (DA) D2 receptor antagonism [36,46,51,52,53]. Liu et al. showed in a mouse model of neuropathic pain that the antinociceptive effects of *l*-THP are abolished with the administration of DA D2 agonists [46]. Similar to other D2 antagonists, *l*-THP has sedative effects at high doses, but the therapeutic effects mentioned above are evident at much lower doses (e.g., <5 mg/kg in rat models of dependence) [32,33,34,35,37,48,54,55,56,57]. Recent studies from the Phillips lab were the first to demonstrate in vivo that *l*-THP modulates DA release in the nucleus accumbens (NAc) via antagonism of the DA D2 autoreceptors [51]. This is particularly intriguing, as it provides a plausible mechanism of action for our observation that *l*-THP reverses withdrawal-associated hypodopaminergia in morphine-dependent rats [51]. We also confirmed the presence of *l*-THP in blood plasma and brain cerebrospinal fluid following treatment with Heantos-4, a botanical formulation used in Vietnam to facilitate opioid detoxification [51]. These findings are part of a growing body of work, both preclinical and clinical, that supports the potential therapeutic utility of *l*-THP in pain and substance use disorders [23,31,49]. However, it has yet to be demonstrated whether *l*-THP is effective in relieving withdrawal-induced pain sensitivity. The main purpose of the current study was to assess the efficacy of *l*-THP in improving pain tolerance during withdrawal. 

The present study was inspired by a recent report by Marchette et al. that a single treatment with the κ-opioid receptor antagonists nor-binaltorphimine or 5′-guanidinonaltrindole resulted in the long-lasting reversal of heroin withdrawal-induced hyperalgesia in male and female rats [13]. We used the core feature of the protocol from Marchette et al. (5 days of opioid treatment followed by 2 drug-free days) to successfully induce robust morphine withdrawal-induced hyperalgesia in rats. Two separate experiments assessed our hypothesis that *l*-THP would reverse this form of hyperalgesia, as assessed by a Von Frey test for mechanical algesia. First, we determined the effects on hyperalgesia during acute withdrawal, when a single dose of *l*-THP was administered 23 h after the previous day’s morphine injection. Second, the outcome of repeated *l*-THP treatment on pain tolerance during detoxification was measured by administering *l*-THP daily during the first 7 days following morphine discontinuation. Finally, we examined the possibility that repeated *l*-THP treatment may have extended effects on prolonged hyperalgesia during the second and third weeks of morphine discontinuation.

## 2. Results

### 2.1. Experiment 1: Hyperalgesia during Acute Withdrawal from Morphine Is Attenuated by l-THP

**Figure 1:**
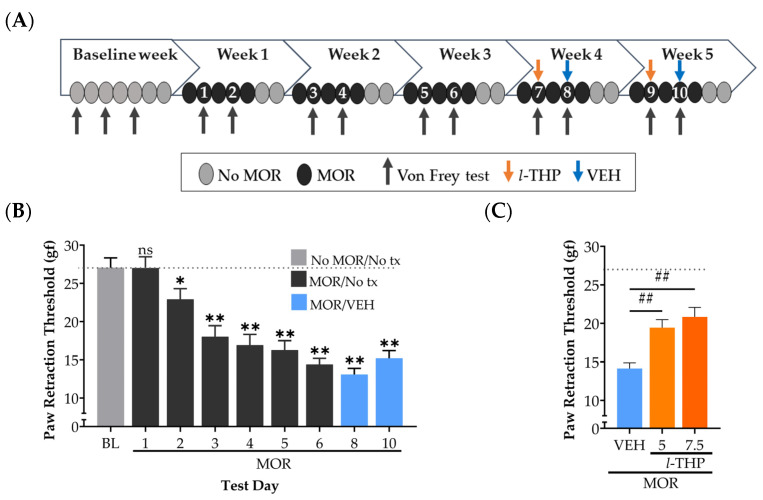
*l*-THP attenuates hyperalgesia during acute withdrawal from morphine (MOR). (**A**) Schedule of MOR injections, *l-*tetrahydropalmatine (*I*-THP) treatments (tx), and Von Frey testing in Experiment 1, where 1–10 refer to Von Frey Test Days. During Weeks 1–5, MOR (15 mg/kg, i.p., *n* = 12) was administered 5 days per week. During Weeks 4 and 5, either *l*-THP (5 or 7.5 mg/kg, p.o.) or vehicle (VEH) was administered 30 min before Von Frey assessments. (**B**) Induction curve of hyperalgesia as indicated by changes in paw retraction threshold at 23 h post-MOR injection. (**C**) Dose-dependent alleviation of MOR withdrawal-induced hyperalgesia by *l*-THP (5 and 7.5 mg/kg, p.o.). The VEH condition is the average of the thresholds obtained after VEH treatment on Test Days 8 and 10 in panel B. Datapoints are the paw retraction threshold (mean + SEM) in grams of force (gf). The dotted line represents the BL threshold value. Dunnett’s test: * *p* < 0.05 and ** *p* < 0.01 vs. baseline (BL). Tukey’s test: ## *p* < 0.01, *l-*THP vs. VEH.

***Hyperalgesia during acute morphine withdrawal.*** This experiment established the time course for inducing withdrawal-induced hyperalgesia in rats receiving morphine for five weeks (Figure 1A). During the week before starting morphine (baseline week), Von Frey tests were conducted on three alternating days to determine the grams of force (gf) required to elicit the paw retraction reflex. There was no significant difference between the three measurements, as indicated by a one-way repeated measures analysis of variance (RM ANOVA; F_2,22_ = 1.180, *p* = 0.326). This indicated that repeated testing did not lead to the development of sensitization or tolerance of the paw retraction reflex. Therefore, the mean of the three threshold values (27.06 ± 1.28 gf) served as the control value in subsequent within-subject analyses. Starting in Week 1, rats received injections of morphine (15 mg/kg, i.p.) 5 days each week for 5 weeks. Bi-weekly Von Frey tests, conducted ~23 h after the previous day’s morphine treatment. The results of these tests confirmed a significant effect of morphine injections on paw retraction threshold (F_8,88_ = 29.75, *p* < 0.001). In comparison to the baseline, the threshold was significantly lower on Test Day 2 (Dunnett’s test, *p* < 0.01) (Figure 1B). From Test Day 4 (16.93 ± 1.41 gf) forward, thresholds persisted at ~40% below baseline until the final assessment on Day 10 (15.21 ± 1.01 gf). These results demonstrated that in our rat model of morphine dependence, a hyperalgesic response to Von Frey tests appears within three cycles of experiencing morphine intoxication and acute withdrawal states. Furthermore, the severity of hyperalgesia was maintained at a consistent level with continued morphine exposure.

***l-THP attenuates hyperalgesia during acute withdrawal from morphine*.** Following three weeks of morphine administration, we assessed the effects of *l*-THP on the paw retraction reflex with either 5 or 7.5 mg/kg (p.o.) administered 30 min before Von Frey assessments on Test Days 7 and 9 in a counterbalanced order (Figure 1A). A one-way ANOVA revealed a significant effect of treatment on paw retraction thresholds (F_2,22_ = 24.74, *p* < 0.001). In comparison to the vehicle, thresholds were significantly higher following treatment with both 5 and 7.5 mg/kg of *l*-THP (Figure 1C) (Tukey’s, *p* < 0.01), representing a 37% and 47% efficacy in attenuating hyperalgesia, respectively. While the improvement was greater following the 7.5 mg/kg dose than the 5 mg/kg dose, there were no statistical differences between the two doses (Tukey’s, *p* = 0.36). Notably, the therapeutic effects of both doses of *l*-THP were limited to the day of treatment. Subsequent Von Frey tests conducted 48 h later under vehicle treatment (Test Days 8 and 10, Figure 1B) indicated hyperalgesia levels comparable to pre-treatment levels (Test Day 6, Figure 1B). 

### 2.2. Experiment 2: Repeated l-THP Facilitates Recovery of Hyperalgesia during Extended Withdrawal from Morphine

**Figure 2:**
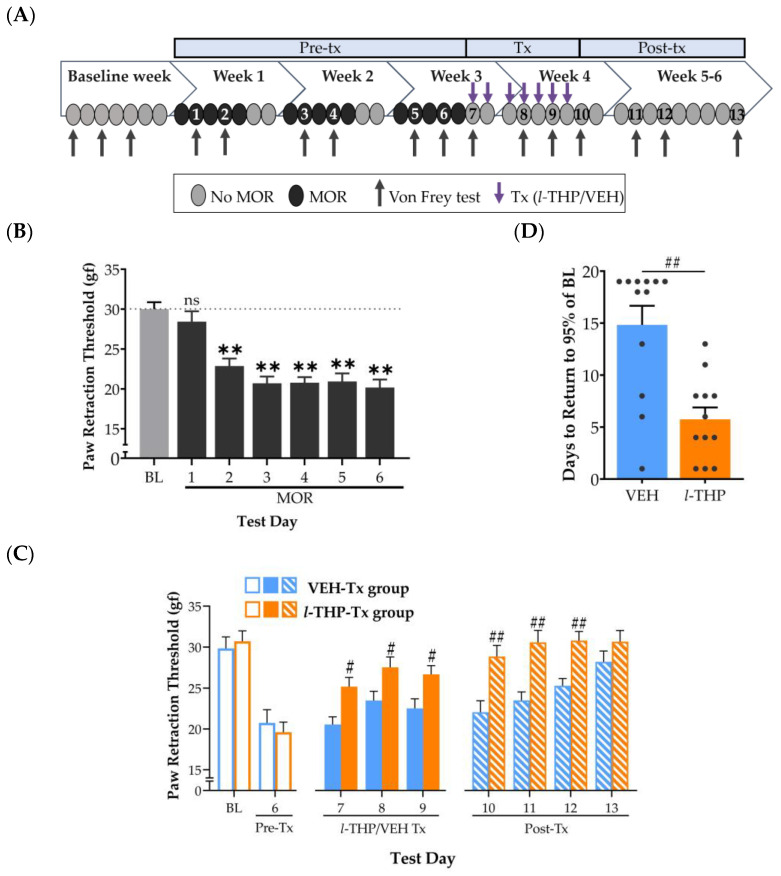
Repeated *l*-THP expedites the recovery of hyperalgesia during extended withdrawal from morphine (MOR). (**A**) Schematic of the MOR injection schedule, *l*-tetrahydropalmatine (*l*-THP) treatments, and Von Frey testing, in Experiment 2, where 1–13 refer to Von Frey Test Days. During Weeks 1–3, MOR (15 mg/kg, i.p.) was administered 5 days a week. *l*-THP (5 mg/kg, p.o.; *n* = 12) or vehicle (VEH, *n* = 12) was administered for 7 days, starting 23 h following the final morphine injection. (**B**) Hyperalgesia during acute withdrawal from MOR, as indicated by Von Frey tests conducted 23 h following the previous MOR injection. The dotted lines represent BL threshold values. (**C**) The alleviation of hyperalgesia during extended withdrawal from MOR following repeated *l*-THP. (**B**,**C**) Datapoints are the paw retraction threshold (mean + SEM) in grams of force (gf). Dunnett’s test: ** *p* < 0.01 vs. baseline (BL). Holm-Sidak: # *p* < 0.05 and ## *p* < 0.01, *l-*THP vs. VEH. (**D**) *l*-THP facilitates the rate of recovery from hyperalgesia during extended withdrawal from MOR. Datapoints represent the number of days to return to 95% of the baseline paw retraction threshold following the final MOR injection. Welch’s *t*-test: ## *p* < 0.01, *l-*THP vs. VEH.

To test the potential for the use of *l*-THP in the treatment of OUD, the second experiment was designed to model detoxification, a critical window of time following the discontinuation of morphine. Rats received weekly morphine injections in the same manner as in Experiment 1, but limited to Weeks 1–3 (Figure 2A). During the baseline week, there was no effect of repeated Von Frey testing on paw retraction thresholds (one-way RM ANOVA: F_2,46_ = 0.68, *p* = 0.511, *n* = 24). Therefore, the mean of the three baseline days (30.27 ± 0.93 gf) was used as the control value in further within-group statistical analyses. During Weeks 1–3, there was a significant effect of MOR injections (15 mg/kg, i.p.) on paw retraction thresholds (one-way RM ANOVA, F_6,138_ = 28.21, *p* < 0.001). The paw retraction thresholds showed a consistent decrease, reaching a plateau of 31–33% reduction from baseline (Figure 2B). These results replicated the findings of Experiment 1, confirming the time course of induction of pronounced hyperalgesia during acute withdrawal from morphine (Figure 1B). 

***Hyperalgesia during extended morphine withdrawal***. During the first week of morphine detoxification (i.e., the treatment period), rats were treated daily with either *l*-THP (5 mg/kg, p.o.; *n* = 12) or vehicle (*n* = 12). Morphine discontinuation did not alter the continued expression of hyperalgesia in the vehicle group (F_13, 143_ = 14.00, *p* < 0.001). The paw retraction thresholds at 24, 96, and 144 h of withdrawal from morphine (21–31% below baseline on Days 7, 8, and 9, respectively) were statistically comparable to those observed following 23 h of withdrawal (30% below baseline on Test Day 6) (Figure 2C). As morphine withdrawal extended into the third week, thresholds in the vehicle-treated group began to approach pre-treatment baseline sensitivity to mechanical stimulation. This demonstrates that in our rat model of morphine dependence, pain tolerance spontaneously recovers over the course of three weeks. 

***Repeated l-THP facilitates recovery from hyperalgesia during extended withdrawal from morphine***. In contrast to vehicle treatment, 7 days of *l*-THP (5 mg/kg, p.o.) administration concurrently with morphine discontinuation had a significant effect on paw retraction thresholds during both the treatment (Treatment Group × Test Day, two-way RM ANOVA: F_3, 66_ = 4.142, *p* = 0.009) and post-treatment periods (Treatment Group × Test Day, two-way RM ANOVA: F_4, 88_ = 2.92, *p* = 0.026). On Test Days 7–9, *l*-THP administration 30 min before Von Frey tests resulted in significantly higher paw retraction thresholds compared to the vehicle-treated group (Holm-Sidak: *p* = 0.03, *p* < 0.05, *p* < 0.05, respectively) (Figure 2C). Importantly, during the post-treatment period, thresholds on Test Days 10–12 were significantly higher in the *l*-THP-treated group than in the vehicle-treated group (Holm-Sidak: *p* < 0.01). This suggests that the pain-alleviating effect of *l*-THP extended significantly beyond its half-life of 4.5 h [58].

We also calculated the number of days required for paw retraction values to return to 95% of baseline values in the morphine-naïve state (i.e., Test Day BL, Figure 2C). There was a significant effect of treatment on days to return to baseline sensitivity to mechanical stimulation (Welch’s *t*-test: t_18.42_ = 4.20, *p* < 0.01). The *l*-THP treatment group showed a significant reduction in the number of days (5.75 ± 1.14) compared to the vehicle group (14.83 ± 1.83) (Figure 2D). These findings were consistent with a faster rate of recovery to pre-morphine pain sensitivity in the *l*-THP-treated group compared to the rate of spontaneous recovery observed in the vehicle-treated group. 

### 2.3. Experiment 3: Effect of l-THP in Morphine-Naïve Rats

***l-THP does not alter pain perception in morphine-naïve rats*.** Here, we assessed the possibility that *l*-THP may have general analgesic properties that could reduce basal sensitivity to mechanical stimulation. In rats (*n* = 11) that received daily saline injections, oral treatment with 5 mg/kg *l*-THP had no significant effect on paw retraction thresholds (33.34 ± 1.91 gf) assessed by Von Frey tests conducted 30 min later, compared to the vehicle treatment (34.85 ± 1.18 gf) (one-way RM ANOVA: F_2,22_ = 0.75, *p* = 0.481). These findings indicate that in morphine-naïve rats, *l*-THP does not have any effect on pain perception in the absence of hyperalgesia. 

### 2.4. Experiment 4: Effect of Withdrawal on Locomotor Activity in Morphine-Dependent Rats

***Morphine withdrawal does not impact locomotor activity in morphine-dependent rats.*** Locomotor activity has been reported to be reduced in various models of neuropathic pain [59,60]. Hence, we examined the possibility that locomotor activity may be altered in our model of morphine dependence. In rats that received 3 weeks of morphine or saline, locomotor activity in an open field was monitored 72 h after the last injection. There was no significant interaction of Treatment Group × Time on the total distance traveled (two-way RM ANOVA: F_1,12_ = 2.288, *p* = 0.16) (Figure S1A). Upon completion of the locomotor test, rats were injected with a final dose of morphine or saline. Twenty-three hours later, there was a significant interaction of Treatment Group × Time on paw withdrawal thresholds (F_1,12_ = 9.115, *p* = 0.01), with morphine-treated rats displaying significantly lower paw retraction thresholds compared to those injected with saline (Holm-Sidak, *p* < 0.01) (Figure S1B). These findings indicate that locomotor activity is not reduced in our model of morphine withdrawal-induced hyperalgesia.

## 3. Discussion

This study established a preclinical model of opioid withdrawal-induced hyperalgesia in morphine-dependent rats using a 5-day-per-week opioid treatment protocol adapted from Marchette et al. [13]. In rats exposed to daily morphine injections, there was a rapid induction and stable expression of mechanical hyperalgesia, similar to that reported by Marchette et al. in heroin-dependent rats [13]. Moreover, we presented the novel finding that animals continued to exhibit hyperalgesia following morphine discontinuation. Indeed, the hyperalgesia persisted for more than 10 days, with a gradual recovery to baseline levels of pain tolerance in the final days of the abstinence period. These findings were consistent with clinical observations that former opioid users continue to exhibit heightened pain sensitivity long after completing detoxification, despite experiencing improvement relative to the degree of hyperalgesia measured during the acute withdrawal period (12–72 h) [10]. The replicability and face validity of this preclinical model of hyperalgesia demonstrate its utility in identifying non-opioid treatment options for the effective management of pain during opioid detoxification. 

The botanical compound *l*-THP, initially derived from *Stephania Glabra* and *Corydalis Yanhusuo,* is used extensively in traditional herbal medicines throughout China and Southeast Asia [50]. As noted in the introduction, both *l*-THP and its parent extracts have been used successfully to treat many forms of acute pain [30], along with the alleviation of hyperalgesia in mouse models of chronic neuropathic and inflammatory pain [47]. Here, we report that *l*-THP (5 and 7.5 mg/kg) given during morphine withdrawal significantly attenuated hyperalgesia (Figure 1). Even at the higher 7.5 mg/kg dose, animals did not show complete recovery to pre-morphine baseline levels, which may reflect the moderate doses used to avoid the possible confounding effect of sedation. Notably, higher doses (10–15 mg/kg) attenuate the behavioral effects of methamphetamine [61]. The apparent increase in paw retraction thresholds observed in our study is not attributed to the locomotor effects of *l*-THP since prior studies report that acute or chronic doses of over 9 mg/kg did affect open field test performance [39,61]. Moreover, our assessment of locomotor activity during 72 h withdrawal demonstrates no impairment in locomotion in animals displaying increased pain sensitivity (Figure S1). This suggests that morphine withdrawal-induced hyperalgesia does not affect locomotion. Overall, our findings provide further confirmation of the analgesic potential of *l*-THP, previously demonstrated in animal models of neuropathic pain [20,45,46,47]. Furthermore, our study extends this effect to opioid withdrawal-induced hyperalgesia, which is clinically relevant to the effective management of opioid detoxification.

Importantly, our findings indicate that repeated dosing of *l*-THP may facilitate detoxification by significantly improving the time to recovery of normal pain perception during abstinence. In animals subjected to a 7-day course of *l*-THP treatment concurrently with morphine discontinuation (Figure 2), hyperalgesia is significantly attenuated during the treatment week. Furthermore, this improvement persists for the duration of the study, with a significant reduction in the number of days to recover to baseline pain threshold scores. Our results provide evidence of the analgesic properties of *l*-THP in OUD, distinct from a recent study by Alhassen et al. confirming the efficacy of *l*-THP in preventing morphine tolerance or OIH, another critical dimension of OUD [41]. Together, these findings add to a growing body of work, both preclinical and clinical, that supports the therapeutic potential of *l*-THP in substance use disorders [23,31,49]. Future studies should include lower doses of *l*-THP to establish a more complete dose-response curve of its analgesic effect on morphine withdrawal-induced hyperalgesia.

*l*-THP itself does not possess intrinsic rewarding or aversive properties, as it does not produce conditioned place preference or conditioned place aversion, respectively [38]. However, it has been shown to successfully blocks drug reward, while inhibiting the acquisition and expression of morphine-induced conditioned place preference [38]. Additionally, *l*-THP has demonstrated the ability to reduce craving and drug-seeking behaviors associated with substances such as heroin, cocaine, ethanol, and nicotine [34,37,42,54,55]. Interestingly, very low doses of *l*-THP, in combination with the opioid antagonist naltrexone, show a synergistic effect in reducing cocaine-seeking in rats [34]. The current study elaborates on the behavioral substrates that may be involved in the relapse-preventing properties of *l*-THP [32,33,34,35,37]. However, future inquiries into the ability of *l*-THP to mitigate the motivating properties of morphine and aversive states during withdrawal will further our understanding of its effects in OUD treatment. Adding to the established body of literature [30,37,38,39,40,41], the present findings suggest that *l*-THP could be a valuable non-opioid pain management option during and after opioid detoxification. These findings suggest that *l*-THP could potentially contribute to the successful long-term management of OUD in clinical populations. 

The present findings that *l*-THP can attenuate morphine withdrawal-induced hyperalgesia are also relevant to the clinical use of Heantos-4, an herbal formulation developed in Vietnam as a supplement to facilitate opioid detoxification. A series of preclinical studies confirm that Heantos-4 has significant effects on DA function in the NAc in morphine-dependent rats while also attenuating somatic signs associated with naloxone-precipitated withdrawal [51,62]. Importantly, *l*-THP is present in both plasma and brain cerebrospinal fluid following oral administration of Heantos-4 [51]. Treatment of morphine-dependent rats with either Heantos-4 or *l*-THP reverses the hypodopaminergia observed in the NAc after naloxone-precipitated withdrawal, along with behavioral signs of withdrawal [51]. It is also of interest that both *l*-THP and Heantos-4 reverse hypodopaminergia induced in the NAc by the DA D2 agonist quinpirole, implicating the DA D2 autoreceptor as their target [51]. This is particularly intriguing, as it suggests a plausible mechanism of action for the reversal of withdrawal-associated hypodopaminergia by *l*-THP [51]. 

These data also take on added significance given recent evidence that DA D1 and D2 receptors are involved in the analgesic effects of *l*-THP in a preclinical model of neuropathic pain. Liu et al. found that *l*-THP has antinociceptive effects in a mouse model of neuropathic pain, an effect that is blocked following the administration of a DA D1 receptor antagonist or DA D2 receptor agonist [46]. Additionally, *l*-THP inhibited overexpression of immediate early genes in the cingulate cortex and periaqueductal grey, which are brain regions involved in physical and emotional pain perception, highlighting these areas as the likely effectors of the antinociceptive effects of *l*-THP [46]. The involvement of dopaminergic signaling in pain perception is not limited to the areas highlighted by Liu et al. Recent optogenetic studies demonstrated that activation of dopaminergic inputs from the ventral tegmental area into the medial prefrontal cortex reduce pain behaviors, further highlighting the role of DA signaling in hyperalgesia [63].While our study presents a novel behavioral pharmacological evaluation of *l*-THP, further studies on the neural substrates of withdrawal-induced hyperalgesia and the mechanisms of the antinociceptive effects of *l*-THP are warranted. 

Human and animal studies confirm the importance of biological sex in OUD, with estrogen-DA interactions enhancing the vulnerability of females to addiction [64,65]. Although there is limited preclinical research specifically focused on sex differences [64], the available data show that female rats are more sensitive to the reinforcing effects of opioids. They acquire higher levels of heroin self-administration and demonstrate greater responsiveness during extinction testing [64,66]. Female rats also display more pronounced and protracted somatic withdrawal signs during abstinence [67,68]. Furthermore, female rats require higher doses of heroin to achieve similar levels of analgesia and experience withdrawal-induced hyperalgesia [13]. Moreover, sex differences in the metabolism of morphine may contribute to the attenuated analgesia observed in females [69]. Using male subjects is a notable limitation of this study, and we recognize the importance of expanding the model presented here to evaluate the efficacy of *l*-THP in attenuating hyperalgesia in female rats. This is particularly important in light of the finding by Marchette et al. that there are sex differences in the efficacy of k-opioid receptor antagonists in attenuating heroin withdrawal-induced hyperalgesia [13]. In future studies investigating the effect of *l*-THP on withdrawal-induced hyperalgesia, careful attention must be paid to determining the dose of morphine required to induce dependence in female rats. Additionally, potential sex differences in the dose range of *l*-THP required to ameliorate this condition should be anticipated and taken into consideration. 

## 4. Materials and Methods

### 4.1. Animals

Sixty-two male Sprague-Dawley rats (250–275 g) from Charles River Laboratories (St. Constant, QC, Canada) were acclimated to the facility for a week prior to any manipulations. Animals were pair-housed in Optirat Cages (Animal Care Systems, Centennial, CO, USA) and maintained with ad libitum access to food and water at ~21 °C in a reverse 12-h light/dark cycle (lights on at 7 p.m.). All experimental procedures were conducted in accordance with the ethical standards set by the Canadian Council on Animal Care and approved by the University of British Columbia Animal Care Committee (AUP A21-0235).

### 4.2. Drug Preparation and Administration

Morphine sulfate from Unipharm Wholesale Drugs Limited (Richmond, BC, Canada) was prepared in saline (15 mg/mL) 1 h before treatment. For 3–5 weeks, animals received a daily injection of 15 mg/kg (i.p.) for five days. This was followed by no morphine for two days each week, an injection schedule adapted from a heroin withdrawal-induced hyperalgesia study by Marchette et al. [13]. This approach incorporates extended (72 h) withdrawal periods to model intermittent morphine access that is associated with increased morphine-evoked hyperdopaminergia in the NAc [70] and more pronounced withdrawal hyperalgesia [71]. 

*l*-THP, purchased from Santa Cruz Biotechnology (Dallas, TX, USA), was dissolved in 0.1 M sulphuric acid and diluted with sterile water to a final concentration of 2.5 mg/mL in 5% acid (pH 4). *L*-THP (5 or 7.5 mg/kg, p.o.) or its vehicle was administered by oral gavage 30 min before behavioral testing. Prior to treatment, animals were habituated to the oral gavage method for 3 days. 

### 4.3. Assessment of Hyperalgesia with Von Frey Device

Hyperalgesia during morphine withdrawal was evaluated using an electronic hand-held Von Frey device, software version 3.4 (Ugo Basile, Gemonio, Italy). Before assessments began, animals were transported to the testing room to acclimate in their home cages for 30 min a day for 3 days. The following week, baseline measurements of sensitivity to mechanical stimulation were assessed on three non-consecutive days (Figure 1A). Each day, animals were brought to the testing room and, after 30 min, placed into the testing apparatus, which consisted of an elevated platform with six Plexiglas compartments (27 cm × 16 cm × 13 cm) and stainless–steel fine-mesh flooring. After 15 min, the Von Frey device was used to apply increasing grams of force to the mid-plantar area of the hind paw until paw retraction was elicited. Each day, six paw withdrawal thresholds were recorded for each subject (three trials per left and right paw), with at least 30 s elapsed between attempts. Any measurements taken concurrently with walking, jumping, and grooming were discarded, and the trials were repeated. 

### 4.4. Assessment of Locomotor Activity in Open Field Test

Locomotor activity was recorded in a Plexiglas open field box (40 cm × 40 cm × 40 cm) with a top-mounted camera (StreamCam, Logitech, CA, USA) for 60 min. All the videos were analyzed by the digital monitoring software Ethovision 11.0 (Noldus, VA, USA). 

### 4.5. Experiment 1

Rats (*n* = 12) were administered morphine injections (15 mg/kg, i.p.) as described above for five weeks. After starting morphine injections, Von Frey tests were conducted as described above on the 2nd and 4th days of each week, ~23 h after the previous morphine injection (Figure 1A). The timing of the test was chosen to capture the peak of withdrawal severity, which occurs at approximately 24 h post-morphine, as reported by Brewer et al. [72]. The effect of *l*-THP on hyperalgesia was evaluated on the 2nd day of Week 4, with half the animals receiving 5 mg/kg and the other half receiving 7.5 mg/kg 30 min before Von Frey testing. During Week 5, rats received an alternate dose of *l*-THP. On the 4th day of Weeks 4 and 5, all animals received vehicle treatment. 

### 4.6. Experiment 2

Following the measurement of baseline values in drug-naïve rats (*n* = 24), subjects were injected once daily with morphine (15 mg/kg, i.p.) during Weeks 1–3 as described above (Figure 2A). Von Frey thresholds were assessed bi-weekly throughout Weeks 1–3 on the 2nd and 4th days of each week, after a 23-h withdrawal from the previous morphine injection. After completing 3 weeks of morphine, subjects entered the abstinence period. On week 4, rats received either *l*-THP (5 mg/kg, p.o.) or vehicle for 7 days. Von Frey testing was conducted 30 min after the 1st, 4th, and 6th treatments, as well as 23 h after the 7th delivery of either *l*-THP or vehicle. Finally, Von Frey assessments continued into Weeks 5–6 in the absence of any further drug interventions. 

### 4.7. Experiment 3

Rats (*n* = 11) were administered saline (1 mL/kg, i.p.) 5 days per week for 2 weeks and assessed for Von Frey thresholds biweekly. Following baseline measures of paw retraction thresholds in an initial test, subsequent assessments were preceded by the administration of either vehicle or 5 mg/kg *l*-THP 30 min before the tests.

### 4.8. Experiment 4

Rats (*n* = 14) were assessed for locomotor activity following the establishment of morphine withdrawal-induced hyperalgesia by administering either saline (control, *n* = 6, 1 mL/kg, i.p.) or morphine (*n* = 8, 15 mg/kg, i.p.) 5 days per week for 3 weeks, as described in Figure 1A. Seventy-two hours after the previous saline/morphine injection, rats were placed in the open field box for 1 h to assess locomotor activity during withdrawal. One more saline/morphine injection was administered after the locomotor test, followed by a Von Frey measurement 23 h later to assess pain sensitivity during withdrawal. 

### 4.9. Data Presentation and Statistical Analysis

Paw withdrawal thresholds, expressed in grams of force (gf) required to elicit paw withdrawal, are presented as a mean of six measurements obtained during each Von Frey test day. Within-subject comparisons of paw retraction thresholds against baseline were conducted using one-way RM ANOVA followed by Dunnett’s multiple comparisons. Tukey’s post-hoc test was used for within-group comparisons of treatment conditions (Figure 1C). Between-group comparisons were performed using a two-way RM ANOVA followed by Holm-Šídák’s multiple comparisons. The box plot method of outlier analysis was applied to mean paw withdrawal thresholds, and one animal identified as an outlier was excluded from the analysis in Experiments 3 and 4. Locomotor activity data in Experiment 4 are presented as a sum of distance traveled in 30 min bins.

Days to return to 95% of BL were determined as the first instance since entering morphine abstinence on which an individual animal displayed a paw retraction response equal to or greater than 95% of baseline thresholds. Animals that failed to reach baseline by the last test day were given a score of 19 days, one above the number of morphine-free days in this experiment. A between-group comparison of days to recover to baseline was conducted using an unpaired *t*-test with Welch’s correction. 

GraphPad Prism version 9.5.0 for Windows was used for all statistical analyses and data visualization (GraphPad Software, San Diego, CA, USA, www.graphpad.com).

## Data Availability

Data available upon request.

## Supplementary Materials

The following supporting information can be downloaded at: https://www.mdpi.com/article/10.3390/ijms24108872/s1.

## Abbreviations

DA—dopamine; *l*-ICP—*l*-isocorypalmine; *l*-THP—*l*-tetrahydropalmatine; MOR—morphine; NAc—nucleus accumbens; OIH—opioid-induced hyperalgesia; OUD—opioid use disorder; OWS—opioid withdrawal syndrome; THPB—tetrahydroprotoberberine
