# Supplementary material for: Morphine Withdrawal-Induced Hyperalgesia in Models of Acute and Extended Withdrawal Is Attenuated by l-Tetrahydropalmatine
Source: Int J Mol Sci. 2023 May 17;24(10):8872. doi: 10.3390/ijms24108872 (PMC10218965; doi:10.3390/ijms24108872)
Supplement: Supplementary file 1 [file ijms-24-08872-s001.zip › ijms-2352747-supplementary.pdf]

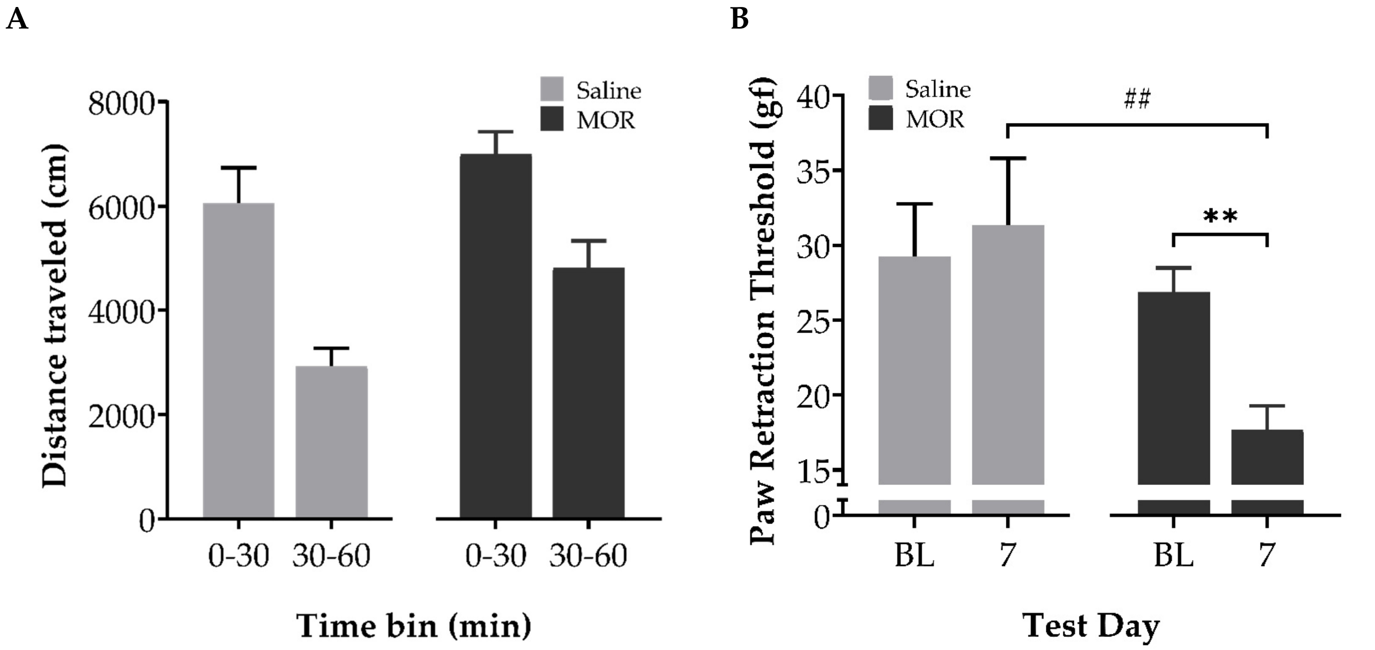

Supplementary Figure S1. Morphine withdrawal-induced hyperalgesia does not impact locomotor activity. Locomotor activity and mechanical sensitivity were measured in animals treated with repeated saline (1 ml/kg, i.p., n=6) or morphine (15 mg/kg, i.p., n=8) for 3 weeks, as described in **Figure 1A**. Von Frey test was performed 23 h after the open field test. **A)** Total distance traveled was recorded 72 h following the previous injection during a 60-min open field test, depicted as two time bins. Datapoints are the total distance (mean+SEM) in centimeters (cm). **B)** Paw withdrawal threshold was recorded on the day following the open field assessment 23 h following the previous injection. Datapoints are paw retraction thresholds (mean + SEM) in grams of force (gf). Holm-Šídák's test: **P<0.01, vs baseline, ##P<0.01 vs saline group.
